# Supplementary material for: Identifying Potential Super-Spreaders and Disease Transmission Hotspots Using White-Tailed Deer Scraping Networks
Source: Animals (Basel). 2023 Mar 26;13(7):1171. doi: 10.3390/ani13071171 (PMC10093032; doi:10.3390/ani13071171)
Supplement: Supplementary file 1 [file animals-13-01171-s001.zip › animals-2179214-supplementary.pdf]

Supplemental Table S1

Scrape ID	Number of Bucks	Scrape Activity	Hotspot Formula	Weighted Degree	Alternative Hotspot Formula	Betweenness
HS1	1.94	0.70	**2.09****	1.20	**2.00****	0.28
HAS	0.30	-0.34	0.06	-1.01	-0.62	-0.89
HS2	1.76	-0.11	0.92	0.90	1.57	0.81
HS4	-0.42	-0.78	-0.38	-0.48	-0.01	-0.89
HS6	**3.22****	0.36	**2.51****	**2.20****	**3.05****	0.62
HS5	1.40	**2.37****	**3.52****	1.53	**2.29****	1.26
HS7	-0.06	-0.57	-0.20	0.09	0.64	-0.19
HS3	0.85	-0.40	0.23	-0.67	-0.24	**3.12****
HSC	-0.79	-0.98	-0.55	-1.15	-0.78	-0.89
FS11	-0.42	-0.24	-0.47	-0.12	-0.45	0.00
FSA	-0.79	-0.36	-0.52	-1.18	-0.84	-0.89
FSB	-0.42	-0.40	-0.48	-1.18	-0.84	-0.89
FS8	-0.42	-0.75	-0.52	-0.28	-0.51	-0.16
FS6	-0.79	-0.67	-0.54	0.35	-0.28	-0.23
FS5	-0.61	-0.36	-0.50	-0.14	-0.46	0.17
FS7	-0.79	-0.47	-0.52	0.13	-0.36	**1.92***
FS9	0.49	0.26	-0.26	**2.04****	0.34	0.00
FS10	-0.06	1.01	-0.25	1.43	0.12	0.00
FS2	-0.42	0.63	-0.38	0.00	-0.41	-0.71
FS1	-1.15	-1.19	-0.57	-0.87	-0.72	-0.89
FS3	0.85	0.70	-0.09	0.90	-0.08	1.01
FS4	-0.42	-0.47	-0.49	0.43	-0.25	**1.68***
RS1	-0.24	-0.24	-0.44	-0.20	-0.34	**1.88***
RS5	0.49	1.12	0.22	1.34	0.44	0.29
RS2	-1.15	-1.11	-0.57	-1.18	-0.84	-0.89
RS7	-1.15	-1.11	-0.57	-1.18	-0.84	-0.89
RS4	-0.06	0.07	-0.26	0.25	-0.11	-0.48
RS3	-0.42	-0.55	-0.45	-0.69	-0.59	-0.78
HSD	-1.15	-1.11	-0.57	-1.18	-0.82	-0.89
GS1	-0.42	-0.40	-0.57	-1.05	-0.85	-0.89
BS1	0.12	3.06	-0.57	-0.75	-0.85	0.40
JS2	1.03	0.57	-0.57	-0.06	-0.85	-0.59
JS1	-0.24	1.81	-0.57	0.59	-0.85	-0.77
